# Optimal Viewpoint Assistance for Cooperative Manipulation Using D-Optimality

**DOI:** 10.3390/s25103002

**Published:** 2025-05-09

**Authors:** Kyosuke Kameyama, Kazuki Horie, Kosuke Sekiyama

**Affiliations:** Department of Mechatronics Engineering, Graduate School of Science and Technology, Meijo University, 501-1 Shiogamaguchi, Nagoya 468-8502, Japan

**Keywords:** cooperative system, optimal viewpoint, mobile manipulator, D-optimality, Fisher information, Next-Best-View, robotic grasping

## Abstract

This study proposes a D-optimality-based viewpoint selection method to improve visual assistance for a manipulator by optimizing camera placement. The approach maximizes the information gained from visual observations, reducing uncertainty in object recognition and localization. A mathematical framework utilizing D-optimality criteria is developed to determine the most informative camera viewpoint in real time. The proposed method is integrated into a robotic system where a mobile robot adjusts its viewpoint to support the manipulator in grasping and placing tasks. Experimental evaluations demonstrate that D-optimality-based viewpoint selection improves recognition accuracy and task efficiency. The results suggest that optimal viewpoint planning can enhance perception robustness, leading to better manipulation performance. Although tested in structured environments, the approach has the potential to be extended to dynamic or unstructured settings. This research contributes to the integration of viewpoint optimization in vision-based robotic cooperation, with promising applications in industrial automation, service robotics, and human–robot collaboration.

## 1. Introduction

Multi-robot systems are advancing to facilitate collaborative information gathering, manipulation, and transportation, allowing robots to complement each other’s functions while executing tasks [1,2]. In particular, real-time information sharing is important, and by each robot collecting and supplementing data, the accuracy of task execution can be improved [3].

In real-world scenarios, robots need to perceive their environment efficiently and cooperatively to obtain more information [4]. Increasing the amount of acquired information plays an important role, for example, in supporting manipulation tasks and understanding the situation in more detail [5]. The basis for obtaining such information is vision-based sensing, and the choice of camera angles and viewpoint has a significantly impact on object recognition performance [6,7]. Therefore, research on optimal viewpoint selection for monitoring robots has proposed methods to determine the optimal camera arrangement to maximize the information gain in reconstructing the environment and object models [8]. Selecting an appropriate viewpoint provides more detailed environmental information and supports the decision-making process of the working robot.

The problem of optimizing the viewpoints of a robot camera can be broadly divided into two categories.

The first category is concerned with reconstructing an unknown environment model by comprehensively capturing the entire environment while minimizing exploration costs. Reily et al. proposed a method to efficiently reconstruct the environment with minimal viewpoints using Transformers, achieving high accuracy recognition [9]. In a multi-robot environment, it is necessary to optimize recognition while reducing redundancy. To this end, Ren et al. proposed an optimization method that integrates viewpoint selection and feature selection to realize collaborative visual recognition [10]. However, these studies focus on reconstructing the environment model and optimizing viewpoint selection and do not directly address specific task execution. Therefore, it is necessary to discuss how a robot makes decisions and executes a task after recognizing the environment.

The second concerns object-centric viewpoint optimization, which determines the position of the robot camera to maximize the information gain from a particular viewpoint. In this context, Guérin et al. proposed a method using numerical optimization to quantify the optimal viewpoint for human pose recognition in 3D space [11]. However, some issues remain regarding consistency with subjective evaluation and applicability to real-world environments. In addition, Kwon et al. used neural networks to learn viewpoint selection that maximizes semantic information acquisition and optimized it using clustering evaluation [6]. This approach enables automatic viewpoint selection and data-driven optimization, but integration with robotic operations remains a challenge. Although these studies contribute to the efficiency of information acquisition, further work is required to verify their effectiveness in real tasks.

In this paper, we address the problem of optimizing the placement of robotic cameras for manipulation tasks in the real world, with a long-term goal of achieving bidirectional viewpoint sharing in multi-robot environments. As a foundational step, we propose a framework for viewpoint optimization of a monitoring robot that provides object shape information in a grasping task. Based on the experimental design framework, we realize the optimal viewpoint selection of the monitoring robot and provide the expected values of the geometric parameters of the object to be grasped, such as the length, width, and height, to the working robot. We use the concept of D-optimality to evaluate the amount of information about the object’s shape from each viewpoint. The value of D-optimality is defined by the determinant of the Fisher information matrix, and a higher value indicates an optimal viewpoint with more information. Furthermore, in order to verify the effectiveness of collaborative work with a monitoring robot, the mobile manipulator performing the grasping task is not equipped with a camera, and the effectiveness of the proposed method is verified in real-world task execution under conditions where available visual information is limited. Experimental results demonstrate that optimal viewpoint selection based on D-optimality improves the accuracy of object shape estimation, highlighting its usefulness for multi-robot manipulation tasks.

## 2. Related Work

Research on optimal viewpoint selection for robots has progressed in two major directions: (1) improving the accuracy of object and environmental recognition/reconstruction, and (2) integrating viewpoint optimization with robot operations in task execution. The former focuses on improving object recognition performance, while the latter has demonstrated the usefulness of viewpoint optimization in a form directly linked to real-world tasks. In the following, we first provide an overview of research aimed at improving recognition accuracy, then examine methods that focus on environment reconstruction and, finally, consider attempts to extend these to actual task execution. Based on the results and challenges of previous studies, we clarify the positioning of this work.

### 2.1. Improving the Accuracy of Object and Environmental Recognition/Reconstruction

As an approach to improve object recognition and class identification, Guérin et al. used a neural network to calculate a semantic score and combine it with the Fowlkes–Mallows index to quantify the amount of information of each viewpoint. This determines the optimal viewpoint, but challenges remain in terms of dataset dependency and adaptation to unknown environments [6]. Kwon et al. proposed a viewpoint optimization method for 3D human pose recognition. In this method, numerical indices such as limb length and visible area are used to reduce computational load while maintaining a high correlation with subjective evaluations. However, since this method is specific to human poses, further research is needed to apply it to more general tasks and dynamic environments [11]. Laga et al. extracted views that facilitate shape classification based on the classification performance of 2D extractions obtained from multiple viewpoints, but challenges remain, such as intra-class variability and the effective use of depth information [12]. Some studies aim to efficiently reconstruct entire environments by controlling the camera viewpoints. Mendoza et al. proposed Next-Best-View with 3D-CNN (NBV-Net) to improve the coverage of object reconstruction without the need for a search process. However, their method is limited to 14 viewpoints, and it remains challenging to deal with continuous viewpoints and dynamic scenes [13]. Potthast et al. developed a probabilistic NBV selection approach using occupancy grid maps to effectively retrieve information even in heavily occluded environments. However, further development is required to improve efficiency and ensure real-time performance under dynamic conditions [14]. Border et al. proposed Surface Edge Explorer, enabling fast and wide coverage by identifying the boundaries of unobserved regions without relying on global scene modeling. However, the applicability of this method to real-world robotic scenarios and its integration into task execution has not been thoroughly verified [15]. In addition, Feixas et al. proposed an information-theoretic approach using mutual information between a set of viewpoints and polygons, enabling a unified treatment of representative-view extraction and saliency estimation in a mesh, but challenges remain in highlighting important regions and adapting to dynamic scenes [16]. Lee et al. used a multi-exposure method to improve the reconstruction accuracy of regions of interest (ROIs) in structured-light cameras, but handling moving objects and coordinating multiple cameras pose further challenges [17].

Efforts to scale up and handle complex problems in viewpoint optimization include research by Morsly et al., who applied particle swarm optimization algorithms to the NP-hard camera placement problem and showed the possibility of a rapid solution. However, further development is required to deal with complex scenarios such as obstacles and real-time requirements [18]. Mantini et al. designed a surveillance camera placement that incorporates human behavior patterns and balances coverage and resolution, but extensions such as simultaneous optimization of multiple cameras and prioritization of specific areas are topics for future research [19].

### 2.2. Integrating Viewpoint Optimization with Robot Operations for Task Execution

On the other hand, research that more directly links viewpoint optimization to task execution includes a framework by Dufek et al. that evaluates the value of viewpoints using Gibson affordances. This approach significantly reduces task execution time and error rates, but real-time adaptation to dynamic tasks and integration with operational planning have not been fully explored [20]. Puligandla et al. proposed an optimization method to maximize the field of view and minimize occlusion, but they mainly focus on extracting multi-resolution candidates without considering coordination with the robot’s motion planning [21]. Cuiral-Zueco et al. introduced a cost function based on the viewing angles and distances of an RGB-D camera to stabilize object tracking and recognition, but did not address optimization for manipulation tasks [22]. Similarly, Ruiz-Celada et al. proposed a smart perception module that integrates object recognition and pose estimation to assist robot motion planning, but real-time viewpoint reconstruction and cooperative task planning remain open challenges [23].

Thus, although research on optimal viewpoint selection has achieved significant results in improving recognition performance and making environment reconstruction more efficient, a framework that fully integrates viewpoint optimization with task planning has not yet been fully established. In viewpoint selection specific to task execution, a major challenge is how to position the camera to improve recognition accuracy when it comes to unknown or unlearned objects.

The technical problem addressed in this study is to minimize the uncertainty associated with the geometric parameters $X=(L_{1},L_{2},L_{3})$, which is equivalent to maximizing the amount of information. We assume that the length, width, and height of the object are equally important. Accordingly, the accuracy of parameter estimation is evaluated based on the Fisher information matrix I(θ). To address this problem, various summary statistics, known as optimality criteria, have been developed to measure how effectively the error ellipsoid can be minimized.

Of these optimality criteria, we focus on A-optimality, E-optimality, and D-optimality. Furthermore, we consider the appropriateness of adopting D-optimality through a comparative analysis with the other optimality criteria. The definitions and characteristics of each are described below.

A-optimality is defined as follows:

(1) $$A=\mathrm{Tr}\left(I{\left(\theta \right)}^{-1}\right)=\mathrm{Tr}\left(\Sigma \right)$$

where I(θ) denotes the Fisher information matrix and Σ represents the covariance matrix of the estimation error. A-optimality aims to minimize the average variance of the estimation error for each parameter. However, this criterion has the characteristic that even if some parameters have large estimation errors, the overall evaluation may still be favorable if the precision for other parameters is sufficiently high. Therefore, for this study, a more uniform accuracy evaluation that does not depend on specific parameters is required.

E-optimality is defined as follows:

(2) $$E=\lambda_{\max}\left(I(\theta )\right)$$

where $\lambda_{\max}$ denotes the maximum eigenvalue of the Fisher information matrix I(θ). E-optimality aims to improve the estimation accuracy in the most uncertain direction by minimizing the maximum eigenvalue corresponding to the direction with the largest variance. This criterion effectively improves the worst-case performance along the eigenvector directions of the covariance matrix (i.e., directions where errors are most likely to spread). However, it does not necessarily improve the accuracy uniformly across all parameters.

D-optimality is an optimality criterion that aims to minimize the volume of the error ellipsoid corresponding to the covariance matrix. It has the property of uniformly improving estimation accuracy in all directions and provides a comprehensive accuracy evaluation that treats all parameters equally. In this study, a detailed optimization method based on D-optimality is developed in the subsequent methodology section.

To deal with this problem, we propose an approach that quantitatively evaluates the utility of viewpoints based on D-optimality, an information-theoretic measure, to build a universal system that does not rely on prior learning of object shapes. Specifically, focusing on the optimal viewpoint selection of a monitoring robot that supports uncertain object recognition, we utilize D-optimality derived from the Fisher information matrix to determine camera configurations that maximize the information gained from each viewpoint.

This enables a statistical processing of object shapes, ensuring versatility for various target objects without depending on existing datasets or pre-trained models. Moreover, our method is designed to be applicable to real-world tasks, thereby enabling the efficient acquisition of information essential for decision-making in manipulation tasks. Ultimately, we aim to deploy our method in multi-robot environments to improve both the success rate and accuracy of tasks and to establish a new framework that integrates task planning and viewpoint optimization.

## 3. D-Optimality-Based Optimal Viewpoint Selection

### 3.1. System Architecture for Cooperative Manipulation Task Execution Utilizing Optimal Viewpoints

**Figure 1:**
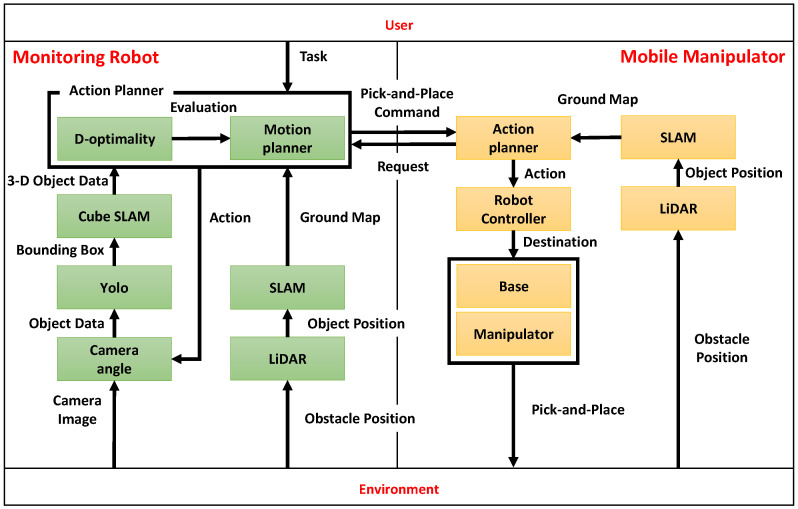
Cooperative system architecture of monitoring robots and mobile manipulators.

In this study, we propose a novel system architecture that enables a mobile manipulator to perform tasks by utilizing optimized object information provided by a monitoring robot. As shown in Figure 1, this system integrates object reconstruction, real-time data processing and information sharing, autonomous optimization of camera placement, and navigation to achieve cooperative manipulation. The proposed task flow aims to grasp, transport, and place an object whose initial position is unknown and which the manipulator itself cannot detect. This is achieved by having a monitoring robot observe a predefined placement candidate area, identify obstacle-free spot within that area, and communicate a safe placement position to the manipulator. To ensure stability and improve task success rate, we propose a system architecture allowing the robot to grasp the object by its shortest edge.

**Figure 2:**
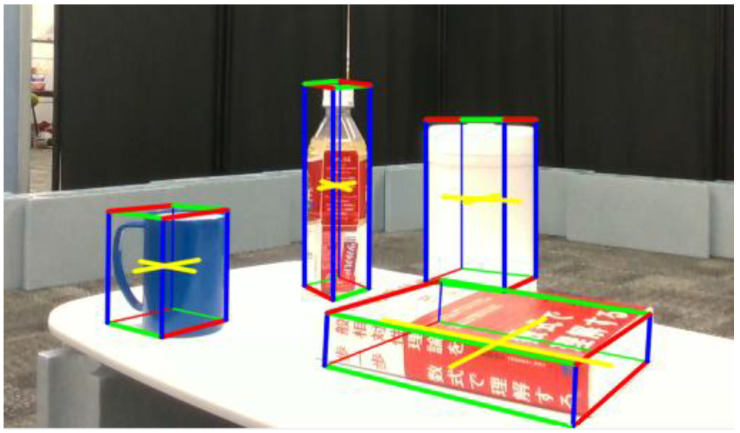
3D(Three Dimensions) Object shape estimation using CubeSLAM.

The monitoring robot is equipped with an RGB-D camera (Intel RealSense D435i, Intel Corporation, Santa Clara, CA, USA) that captures images of the surroundings in real-time. Using the acquired images, 2D object detection is performed with YOLOv5 (v5.0, Ultralytics LLC, Redmond, WA, USA), which identifies the center coordinates of the object based on the bounding box and depth information. Furthermore, as shown in Figure 2, the cuboid approximation function, part of CubeSLAM, is utilized in 3D object reconstruction. Specifically, the 3D shape of the object (length, width, and height) is estimated based on the 2D bounding boxes obtained by YOLOv5 and vanishing points [24]. It should be noted that the SLAM functionality itself is not used in this system, as described in Section 3.2.

In this study, we quantitatively evaluate the viewpoint using D-optimality, based on the geometric parameters (length, width, height) of the estimated object. A higher value of D-optimality indicates a smaller variance in the shape estimation of the target object, and thus a higher estimation accuracy. However, as this value is relative and may vary depending on the environment, a viewpoint with a relatively high D-optimality in a particular environment means a viewpoint that has improved shape estimation accuracy. Therefore, a threshold is set to determine whether D-optimality is comparatively high or low, and viewpoints that exceed this threshold are searched for. Since object recognition performance is highly dependent on the relative angle between the camera and the object, we maintain a distance of 1 m from the object and search for the optimal viewpoint by changing the camera angle by approximately 10 degrees. The distance from the object is fixed at 1 m in order to stabilize visibility and simplify control. The effect of distance changes on recognition accuracy is outside the scope of this study, and is not discussed in this paper. A threshold, determined experimentally in advance, is then used to identify viewpoints exceeding that threshold as the optimal ones, and the resulting object information is shared with the manipulator in real time.

The monitoring robot employs mecanum wheels to move in all directions and uses LiDAR. It also adjusts its orientation based on RGB-D images obtained from the environment to ensure an optimal viewpoint.

The mobile manipulator is equipped with AMIR740 five-axis robotic arm (Vstone Co., Ltd., Osaka, Japan) and supports omnidirectional movement with mecanum wheels. It also performs self-localization using LiDAR, but since it does not have an RGB-D camera, it utilizes real-time object information (center coordinates, shape, and orientation) obtained and shared by the monitoring robot.

When the mobile manipulator grasps an object approximated as a rectangular parallelepiped, it is optimal to select the short side, as this choice provides greater clearance in the end effector’s opening width and increases tolerance for positional offsets, thereby improving the success rate. Since the five-axis mobile manipulator needs to approach the perpendicular line of the short side of the object, it is necessary to set the target point on that perpendicular line at a distance suitable for grasping while avoiding obstacles. The manipulator then executes the grasping task based on this posture.

In addition, two candidate placement locations are preassigned exclusively to the monitoring robot. The monitoring robot is responsible for assessing the occupancy status of each candidate location. Specifically, if no object is detected within a radius of 20 cm from a candidate location, the location is judged as “vacant”. Conversely, if an object is detected within 20 cm, the location is judged as “occupied”. Based on this judgment, the monitoring robot determines the final placement location. It should be noted that this study does not consider the case where both candidate locations are occupied. In contrast, if both locations are vacant, the location that is farther from surrounding objects—i.e., the one with fewer nearby obstacles—is selected as the placement destination.

A configuration in which the manipulator does not obtain information directly from the camera but works in cooperation with an additional monitoring robot is designed to verify the following:

(1) To quantitatively evaluate the information value of each viewpoint based on the accuracy of shape estimation.
(2) To confirm that information obtained from an external viewpoint remains valid even after being shared.

In this configuration, it is assumed that, when a mobile manipulator works independently, there is a possibility that the observation information from its viewpoint alone may be insufficient to perform the task. In such a situation, it becomes necessary for other robots to observe the object from an appropriate viewpoint and share more accurate information. In particular, when the information obtained by the manipulator is highly uncertain, complementary cooperation in the information space is likely to enable more robust perception and decision-making.

It should be noted that if the camera information from both robots were integrated during verification, it would become unclear to what extent each information source contributed. Therefore, in order to clearly evaluate the contribution of the external viewpoint, this paper evaluates under conditions in which the manipulator is not equipped with a camera.

### 3.2. Object Modeling and Coordinate Transformation

CubeSLAM is based on ORB-SLAM2, where the camera pose is estimated by matching ORB feature points between frames. By integrating the camera trajectory with object information, a high-precision 3D map can finally be constructed. However, SLAM using ORB-SLAM2 can be computationally expensive, posing a significant load for real-time performance. Moreover, because feature points must be observed from multiple viewpoints, it becomes necessary to ensure multi-view imaging.

Therefore, in this study, we approximate 3D objects only as rectangular parallelepipeds (cuboids) and estimate the shape and pose of these objects using geometric parameters such as the length, width, and height of the approximated edges. This method makes it possible to treat uncertain object dimensions statistically, thereby reducing the computational load and improving real-time performance.

**Figure 3:**
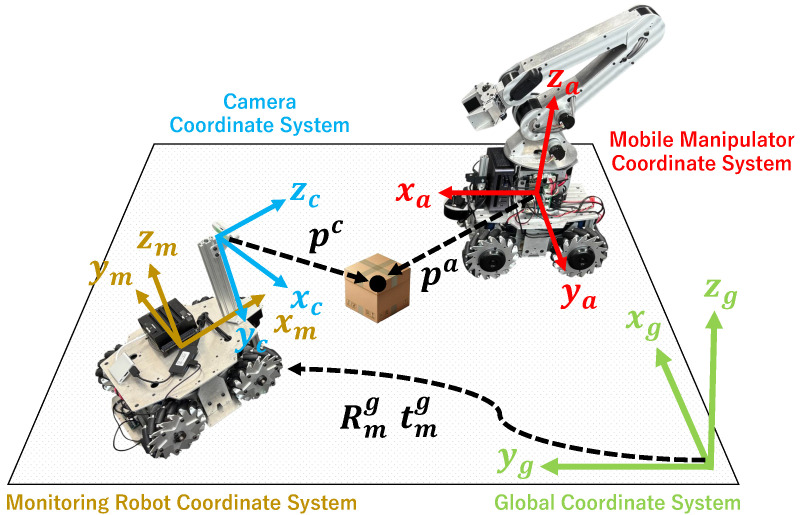
Definitions of the global, robot base, and camera coordinate systems used for coordinate transformation.

The definitions of each coordinate system are shown in Figure 3, and the object information obtained in real-time through CubeSLAM is represented in the camera coordinate system. Consequently, the object position estimated by the monitoring robot needs to be transformed into the manipulator’s coordinate system. The following subsections provide the details of this coordinate transformation.

(1) Transformation from Camera Coordinate System to Monitoring Robot’s Base Coordinate System

Let $p^{c}\in R^{3}$ denote the position of an object in the camera coordinate system. The corresponding position vector $p^{m}$ in the monitoring robot’s base coordinate system is given by

(3) pm=Tcmpc,

where Tcm is a 4×4 homogeneous transformation matrix composed of the rotation matrix and the translation vector defined as

(4) Tcm=Rcmtcm0T1.

Here, $R_{c}^{m}$ is the 3×3 rotation matrix, and $t_{c}^{m}\in R^{3}$ is the translation vector, representing the relative orientation and position between the camera and the base coordinate system of the robot. Since the relative positional relationship between the camera and the robot base is fixed in this study, this matrix remains constant.

(2) Transformation from Monitoring Robot’s Base Coordinate System to Manipulator’s Base Coordinate System

Both the monitoring robot and the manipulator perform their own pose estimation, and the relative relationship between them changes dynamically. Throughout this section, the subscript *m* refers to the monitoring robot, and *a* refers to the manipulator. Let TmG and TaG denote the estimated poses of the monitoring robot and the manipulator in the global coordinate system G, respectively. These 4×4 homogeneous transformation matrices are defined as

(5) TmG=RmGtmG0T1,TaG=RaGtaG0T1.

Here, the rotation matrix $R^{G}\in R^{3\times 3}$ and the translation vector $t^{G}\in R^{3}$ can be expressed in general form as

(6) $$R^{G}=\left[\begin{array}{ccc} r_{11} & r_{12} & r_{13}\\ r_{21} & r_{22} & r_{23}\\ r_{31} & r_{32} & r_{33} \end{array}\right],\;t^{G}=\left[\begin{array}{c} t_{x}\\ t_{y}\\ t_{z} \end{array}\right].$$

Then, the transformation matrix from the monitoring robot’s base coordinate system to the manipulator’s base coordinate system Tma is given by

(7) Tma=TaG−1TmG.

By using this transformation, the relative positional relationship between the monitoring robot and the manipulator can be obtained in real time.

(3) Overall Transformation

Finally, the object position $p^{c}$ measured in the camera coordinate system is mapped into the manipulator’s base coordinate system as follows:

(8) pa=TmaTcmpc=TmaTcmpc,

where the static transformation matrix Tcm is combined with the dynamically changing matrix Tma. This allows the 3D information obtained in the camera coordinate system to be accurately transmitted to the mobile manipulator. In the cooperative tasks of this study, the accuracy of this coordinate transformation directly affects both the success rate and efficiency of object manipulation, so appropriate pose estimation and matrix calculation are extremely important.

## 4. Optimization of Viewpoints Using D-Optimality

### Derivation of the Multivariate Normal Distribution and D-Optimality

The process of selecting the optimal viewpoint using D-optimality is shown in Algorithm 1. The geometric parameters $X=(L_{1},L_{2},L_{3})$ of the object obtained from each camera pose $\left\{\alpha_{1},\alpha_{2},\ldots ,\alpha_{i}\right\}$ are given as input, and the camera pose $\alpha_{\max}$ with the highest evaluation is selected as the optimal viewpoint. Although the distance to the target object should also be considered as a variable, the effect of distance on recognition accuracy is mainly due to the visibility of the object from the camera, and the trend is relatively constant. Therefore, it is considered unnecessary to estimate the distance separately.
**Algorithm 1:** Threshold-based View angle Selection Using D-optimality
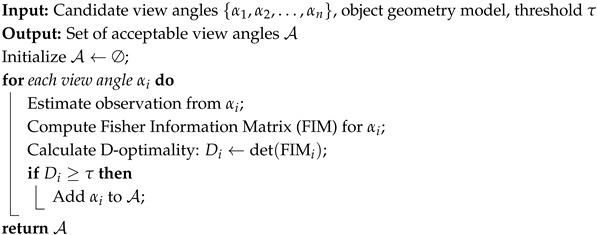


**Figure 4:**
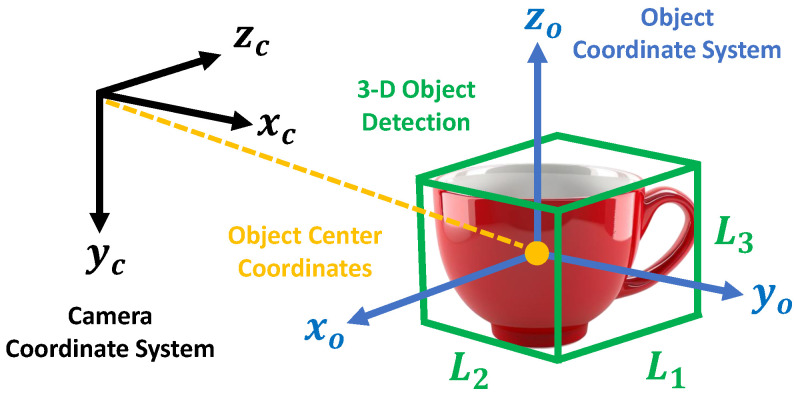
The definitions of the camera and object coordinate systems.

The definitions of the camera and object coordinate systems are illustrated in Figure 4. In this paper, the shape of the target object is represented by a 3D vector $X=(L_{1},L_{2},L_{3})$, which corresponds to the length, width, and height of the object. In this section, we explain the rationale behind our parameter selection. The basic premise is that the key goal of information sharing among multiple robots is not to perfectly reproduce the environment and objects, but to efficiently convey only the information necessary for other robots to complete their tasks.

Therefore, in this study, we do not consider the reconstruction of complex object shapes and we limit the modeling of target objects to symmetric shapes such as plastic bottles or rectangular parallelepiped shapes such as books. As long as the approximation of object shapes does not significantly affect manipulation performance, it is more appropriate to generalize the shape and apply the D-optimization algorithm to a wider range of objects for the purpose of information sharing. An important consideration here is the extent to which shape approximation affects manipulation performance.

However, for symmetrical or rectangular objects, shape approximation errors are minimized, so this is not expected to cause any major problems. Furthermore, length, width, and height are basic parameters commonly used in many object shape representations, so they are suitable for the purpose of this research.

The object coordinate system is defined as a right-handed orthogonal coordinate system whose origin is located at the center of the cuboid and whose axes are aligned with the principal axes of the object (i.e., length, width, and height).

It is important to note that the components of X depend on the orientation of the object at the time of detection, and their order is not guaranteed to be uniquely preserved. In the proposed method, the edge that appears closest to the camera along the positive *Z*-axis of the camera coordinate system is recognized as $L_{2}$. If the object is rotated clockwise by $90^{\circ }$ around the positive *Y*-axis of the camera coordinate system in a right-handed manner, the same edge is then recognized as $L_{1}$.

Therefore, due to variations in object orientation, the order of the components of X may change, and the semantic correspondence of each dimension may not be consistently maintained. Nevertheless, in this study, such inconsistencies in order do not affect the final estimation results.

Let the mean vector be $\mu =(\mu_{1},\mu_{2},\mu_{3})$ and the covariance matrix be Σ. The probability density function of X is given by Equation (9):

(9) $$\begin{array}{cc} f(X|\theta )\; & =\frac{1}{\sqrt{{\left(2\pi \right)}^{k}\;\det\left(\Sigma \right)}}\exp\;\left(-\frac{1}{2}{\left(X-\mu \right)}^{T}\;\Sigma^{-1}\;\left(X-\mu \right)\right), \end{array}$$

where θ=μ and k=3. The covariance matrix Σ is defined as follows:

(10) $$\begin{array}{cc} \Sigma & =\left[\begin{array}{ccc} \sigma_{1}^{2} & \mathrm{Cov}(L_{1},L_{2}) & \mathrm{Cov}(L_{1},L_{3})\\ \mathrm{Cov}(L_{2},L_{1}) & \sigma_{2}^{2} & \mathrm{Cov}(L_{2},L_{3})\\ \mathrm{Cov}(L_{3},L_{1}) & \mathrm{Cov}(L_{3},L_{2}) & \sigma_{3}^{2} \end{array}\right], \end{array}$$

Here, $\sigma_{i}^{2}$ is the variance of $L_{i}$, and $\mathrm{Cov}(L_{i},L_{j})$ is the covariance between $L_{i}$ and $L_{j}$. Because the measurement error variance ($\sigma_{i}^{2}$), due to sensor hardware characteristics, is assumed to be constant, this study estimates only the parameter θ=μ. Taking the logarithm of Equation (9) yields the log-likelihood function as follows:

(11) $$\begin{array}{cc} \log f(X|\theta )\; & =-\frac{1}{2}\log\;\left({\left(2\pi \right)}^{k}\;\det\left(\Sigma \right)\right)-\frac{1}{2}{\left(X-\mu \right)}^{T}\;\Sigma^{-1}\;\left(X-\mu \right), \end{array}$$

The Fisher information matrix I(θ) is an indicator of the sensitivity of parameter estimation. Based on Equation (11), it is defined by taking the expected value of the gradient concerning μ as follows:

(12) $$\begin{array}{cc} I(\theta )\; & =E\;\left[\left(\frac{\partial }{\partial \mu }\log f\right){\left(\frac{\partial }{\partial \mu }\log f\right)}^{T}\right]=\Sigma^{-1}, \end{array}$$

where $\Sigma^{-1}$ is also called the precision matrix. Finally, D-optimality is an optimality criterion that maximizes the determinant of the Fisher information matrix. Using Equation (12), D-optimality is defined by

(13) $$\begin{array}{cc} D & =\det\;\left(I(\theta )\right)=\det\;\left(\Sigma^{-1}\right)=\frac{1}{\det\;\;\left(\Sigma \right)}. \end{array}$$

A higher value of D-optimality indicates a smaller covariance matrix Σ. Hence, the viewing angle α that maximizes D-optimality corresponds to the configuration in which the variance in estimating the shape of the target object is minimized—in other words, the configuration with the highest estimation accuracy.

## 5. Verification of Cooperative Manipulation System Using Viewpoint Optimization Based on D-Optimality

### 5.1. Verification of Object Estimation Accuracy Using D-Optimality

#### 5.1.1. Experimental Environment

**Figure 5:**
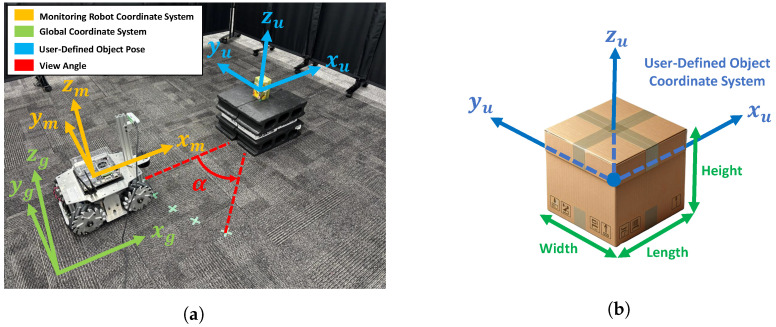
Experimental environment: (**a**) The monitoring robot, global coordinate system, and user-defined object coordinate systems. (**b**) Details of the user-defined object coordinate system.

Through this experiment, we evaluate how the 3D shape estimation results of a target object observed by the monitoring robot relate to D-optimality. Figure 5a shows the global coordinate system in the experimental environment, the monitoring robot coordinate system, the user-defined object coordinate system, and the viewing angle. Figure 5b shows the details of the user-defined object coordinate system. The object coordinate system shown in Figure 4 is defined in the camera coordinate system based on the estimated cuboid. However, since this coordinate system is derived from the estimated rectangular parallelepiped seen from the camera, it does not necessarily match the shape or edge direction of the actual object. Therefore, in this study, we define a new right-handed Cartesian coordinate system with the center of the actual object as the origin and each axis aligned with the main directions (length, width, height) of the object. This coordinate system is referred to as the user-defined object coordinate system.

By introducing the user-defined object coordinate system, it becomes possible to accurately define and compare the angular difference (rotation around the Z-axis) between the monitoring robot and the object in a coordinate system that reflects the actual shape and orientation of the object. By focusing on the Z-axis rotation angle α between the monitoring robot coordinate frame and the object, the relationship between the robot’s observation direction and the target’s principal axis can be quantitatively evaluated.

The object used in this experiment is a cuboid with dimensions L×W×H=8cm×7cm×12.5cm. By using a simple cuboid shape, the error between the actual size and the estimated value from CubeSLAM can be more clearly compared. The object was also placed so that its axes were aligned with those of the global coordinate system.

The monitoring robot observes the target object while maintaining a distance of approximately 1 m. The robot’s orientation angle (deflection around the positive direction of the *Z* axis) relative to the object, denoted as α, is set as the control variable, with five different values: $\alpha =0^{\circ },\;10^{\circ },\;20^{\circ },\;30^{\circ },\;40^{\circ }$. At each viewpoint angle, 1000 3D data samples X are obtained from CubeSLAM, and D-optimality is calculated for each sample. Also, the error between the estimated object dimensions and the ground truth is received, and the variation range for each viewpoint is displayed as a box plot. This allows us to quantitatively evaluate whether viewpoints with higher D-optimality values lead to improved estimation accuracy.

To ensure a fair evaluation, the lighting conditions and arrangement of stationary objects in the experimental environment are set to be constant, and a simple layout is adopted to facilitate the identification of the object and robot coordinate systems. Furthermore, the monitoring robot uses LiDAR-based self-localization to move to the target viewpoint and adjust its viewing angle. By maintaining these consistent conditions, we ensure that the relationship between viewpoint angle, D-optimality, and estimation accuracy is purely evaluated.

#### 5.1.2. Experimental Results

**Figure 6:**
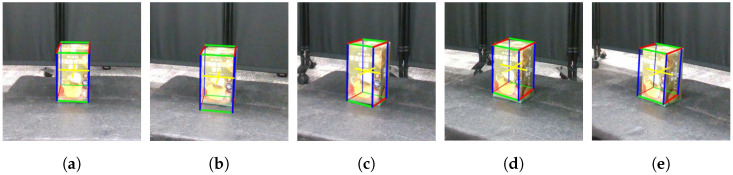
Estimation results of object shapes under different viewing angles: (**a**) $\alpha =0^{\circ }$, (**b**) $\alpha =10^{\circ }$, (**c**) $\alpha =20^{\circ }$, (**d**) $\alpha =30^{\circ }$, and (**e**) $\alpha =40^{\circ }$.

**Figure 7:**
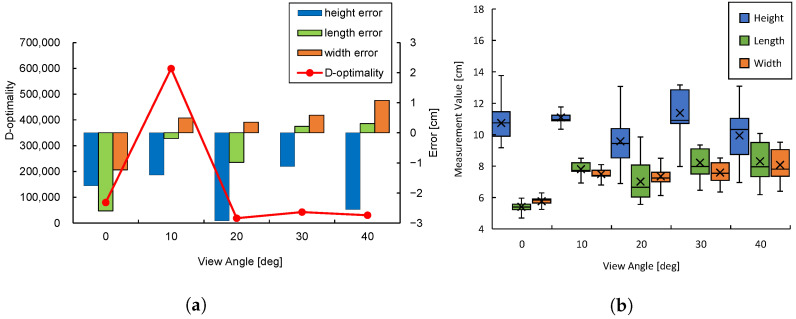
Experimental results on D-optimality and object estimation accuracy: (**a**) D-optimality and the mean error of shape estimation. (**b**) Distribution of estimated object shapes.

Figure 6 shows snapshots of the cuboids estimated by CubeSLAM at each viewing angle. Figure 7a shows the relationship between D-optimality and shape estimation error for each angle. The highest D-optimality value is obtained at $\alpha =10^{\circ }$. Other angles show relatively lower values, suggesting a correlation with estimation accuracy. On the other hand, examining the object estimation error in detail reveals that the blind spots and viewing angles of the sensor make shape estimation unstable, leading to large negative height estimation errors. However, at $\alpha =10^{\circ }$ and $30^{\circ }$, height estimation errors are relatively small, suggesting that certain viewing angles improve estimation accuracy.

Figure 7b visualizes the distribution of estimated data obtained at each angle using box plots. While the mean estimation error for $\alpha =10^{\circ }$ and $30^{\circ }$ is similar, the data distribution at $\alpha =10^{\circ }$ is smaller, with all edges falling within 2 cm, confirming the smaller estimation variance. This result supports the theoretical background of Equation (13), which implies that higher D-optimality corresponds to lower measurement variance. Conversely, at $\alpha =30^{\circ }$, the estimation variance is larger, with the estimated height deviation exceeding 5 cm, indicating increased uncertainty.

Equation (13) defines D-optimality to be minimized when the determinant of the covariance matrix $\det\;\;\left(\Sigma \right)$ is maximized. That is, the smaller the determinant, the smaller the variance of the measured data and the better the estimation accuracy. Indeed, maximizing D-optimality with $\alpha =10^{\circ }$ minimizes the variance of the acquired data and provides stable shape estimation.

From these results, we conclude that there is a clear relationship between D-optimality and estimation error. Particularly, the viewpoint at $\alpha =10^{\circ }$ shows a similar mean error as other angles but with a higher concentration of data leading to more consistent estimates. This finding suggests the effectiveness of using D-optimality for viewpoint selection to enhance shape estimation accuracy.

### 5.2. Improving Manipulation Accuracy Using D-Optimality

#### 5.2.1. Experimental Environment

**Figure 8:**
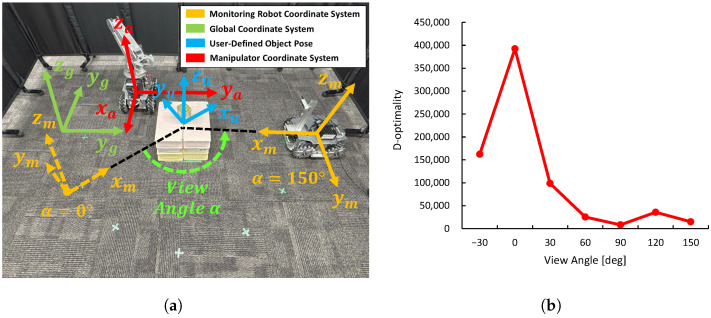
Experimental environment for D-optimality, object center coordinate estimation accuracy, and manipulation accuracy: (**a**) Experimental environment showing coordinate systems of each robot and object ($\alpha =150^{\circ }$). (**b**) Preliminary evaluation of D-optimality at each sampling point.

This experiment aims to evaluate the impact of variations in D-optimality on the estimation of object center coordinates, and manipulation accuracy. Figure 8a shows an overview of the experimental environment, illustrating the coordinate system of the monitoring robot, the mobile manipulator coordinate system, and the object coordinate system. The experimental environment is set up in an indoor space partitioned to minimize external lighting conditions and unnecessary disturbances to the sensors. The monitoring robot and the manipulator each perform self-localization using LiDAR, ensuring that they can continuously recognize each other’s relative coordinate systems.

The target object is a cuboid with dimensions L×W×H=8cm×7cm×12.5cm. In this experiment, it is placed at (x,y,z)=(50.0cm,40.0cm,33.0cm) in the base coordinate system of the manipulator. This placement was selected to ensure that the robot can easily grasp the object.

As shown in Figure 8a, the monitoring robot maintains a constant distance of 1m from the object while adjusting the inclination angle α, which corresponds to rotation around the positive Z-axis of the camera. Specifically, seven different angles $\alpha =0^{\circ },30^{\circ },60^{\circ },90^{\circ },120^{\circ },150^{\circ },180^{\circ }$ are set, and a preliminary evaluation of D-optimality is conducted for each viewpoint. The viewpoint angle α is adjusted by controlling the orientation of the camera via the wheel drive of the monitoring robot.

The base coordinate system of the manipulator is fixed, and the target object is placed at a location where grasping is possible without moving the base. The mobile manipulator executes the grasping task based on the position information provided by the monitoring robot. The maximum gripping width of the end effector is 9 cm, while the edge of the grasping object is 7 cm.

In the preliminary evaluation of D-optimality at each viewpoint, 1000 sets of 3D object data X are collected, and D-optimality is calculated. Previous experimental results have shown that viewpoints with higher D-optimality yield more accurate estimates of object shape. Based on this, it is expected that the estimation accuracy of the object center coordinates and the resulting manipulation accuracy will improve. To verify this, the results of the preliminary evaluation of D-optimality in the experimental environment are shown in Figure 8b. The viewpoint with the highest D-optimality is when $\alpha =30^{\circ }$, while the viewpoint with the lowest D-optimality is when $\alpha =120^{\circ }$. From these two viewpoints, the monitoring robot transmits the estimated object center coordinates to the manipulator, which then performs the grasping task. This trial is repeated 10 times for each viewpoint. To evaluate the grasping accuracy, the difference between the actual tip position of the end effector and the ideal object center position is measured and the number of successful grasps is recorded to compare grasping success rate and demonstrate the effectiveness of D-optimality in manipulation tasks.

A quantitative analysis was conducted using the following indicators:

(1) The accuracy of grasp position estimation was evaluated in the perception phase, prior to object grasping. The mean error was calculated based on the Euclidean distance between the estimated object center and the true object center. This is a quantitative indicator of how much the D-optimality algorithm contributes to the accuracy of object position estimation.
(2) The variance of the estimation results across multiple trials was compared to evaluate the stability and reproducibility of the task. This confirms that the viewpoint selection not only improves the success rate in a single trial but also consistently achieves a good success rate.

Through these evaluations, the validity and effectiveness of the proposed method are further verified.

#### 5.2.2. Experimental Results

In this study, we hypothesize that higher D-optimality, as defined by Equation (13), leads to reduced measurement variance, improving object shape estimation accuracy and consequently enhancing manipulation accuracy. In fact, measurements obtained from viewpoints with the highest D-optimality exhibit smaller errors relative to the ground truth (actual object center coordinates), whereas measurements from viewpoints with lower D-optimality show larger estimation errors.

**Figure 9:**
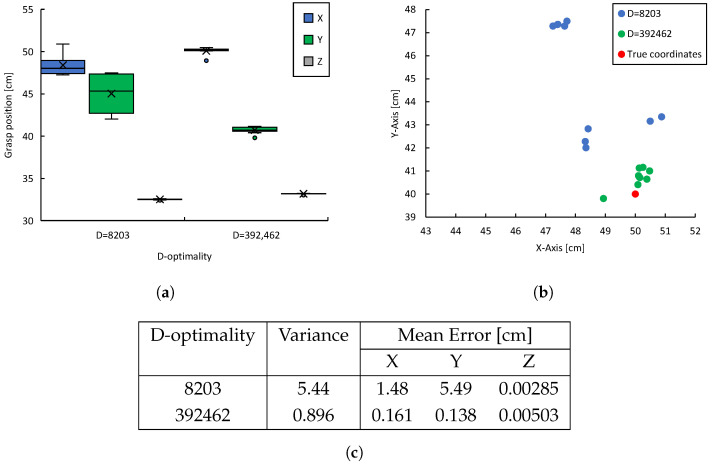
Experimental results concerning D-optimality, object center position estimation accuracy, and manipulation accuracy: (**a**) Distribution of the manipulator’s grasping positions. (**b**) Distribution of grasping positions in the X-Y plane. (**c**) Comparison of the variance and mean error of grasp positions.

Figure 9a summarizes the grasping positions of the manipulator for the highest and lowest D-optimality viewpoints in the preliminary evaluation. The improved shape estimation accuracy reduces the estimation error of the object center coordinates. Consequently, the alignment accuracy of the manipulator with the grasping target improves, leading to reduced variation in manipulation. As shown in Figure 9b, the variance in grasping positions across multiple trials is smaller for viewpoints with high D-optimality. In 10 trials, grasping success was achieved 0 times at the viewpoint with D=8203, while at the viewpoint with *D* = 392,462, 8 successful grasps were achieved, confirming the tendency for improved manipulation task accuracy. As shown in Figure 9c, these results show the variance and mean errors of object center estimations obtained from viewpoints with different levels of D-optimality. For low D-optimality (8203), the variance of the estimation results was 5.44, and the mean errors were 1.48cm in the X direction, 5.49cm in the Y direction, and 0.00285cm in the Z direction. In contrast, for high D-optimality (392,462), the variance was reduced to 0.896, and the mean errors were significantly reduced to 0.161cm, 0.138cm, and 0.00503cm in the X, Y, and Z directions, respectively.

These results indicate that selecting a viewpoint with higher D-optimality reduces the variance of the estimation results by approximately one-sixth and improves the positional estimation accuracy in the X and Y directions by factors of about nine and forty, respectively. These results strongly indicate that viewpoint selection is effective in improving recognition accuracy, especially the significant reduction in error in the Y direction. It is noteworthy that the estimation error in the Z direction remained very small in both conditions, indicating that viewpoint selection has a relatively small effect on accuracy in the Z direction.

In summary, these findings quantitatively confirm that viewpoint selection based on high D-optimality improves both estimation accuracy and task stability.

In particular, by reducing the estimation error of the object center coordinate, the stability of the end effector grasping operation is improved. Below, we will explain the two failure cases in detail. The first failure occurred during a grasping attempt at (x,y,z)=(48.9cm,39.8cm,33.2cm), and the second occurred at (x,y,z)=(50.1cm, 40.7cm, 33.1cm). Although both positions are within a small margin of error from the true object position (x,y,z)=(50.0cm, 40.0cm, 33.0cm), the grasps were unsuccessful. These results indicate small estimation errors of the object center do not necessarily guarantee a successful grasp.

The failed manipulation attempts are considered to have been caused by slight discrepancies in the initial poses of the robot and the target object. These errors are unrelated to the shape estimation results obtained through viewpoint selection and are failure factors caused by the manipulator itself that cannot be avoided by visual support alone. The effectiveness of the proposed method is primarily limited to reducing uncertainty at the perception stage and does not guarantee the precision of motion planning or control execution. Therefore, these failures do not negate the validity of the D-optimality algorithm itself. Such issues can be overcome by improving the accuracy of coordinate transformations or by introducing cooperative control algorithms. However, because the system design in this study handles viewpoint selection independently, such errors are structurally likely to occur and are a disadvantage brought about by the system. The effectiveness of the proposed method is based on the assumption that the robot’s control system is constructed with a certain degree of accuracy. The accuracy required for operation depends on factors such as the robot’s self-localization accuracy and calibration accuracy, so the allowable error must be flexibly adjusted according to these conditions.

Based on these experimental results, the effects of high D-optimality viewpoints can be summarized as follows:

(1) Improved Shape Estimation Accuracy: A viewpoint with high D-optimality reduces the variance of shape-related data and reduces the estimation error of object dimensions. Consequently, the object center coordinates can be determined more accurately.
(2) Improved Accuracy of Grasping Position: By improving the accuracy of shape and center coordinate estimation, deviation from the target grip position is suppressed. As a result, variations in grasping positions are minimized, enabling more consistent grasping at positions close to the true value.
(3) Effectiveness of D-optimality as an Index: Experimental results show that there is a clear correlation between D-optimality and estimation and grasping accuracy—that is, selecting a viewpoint with high D-optimality obviously improves the success rate and accuracy of the task execution.

### 5.3. Collaborative Manipulation Through Optimal Viewpoint Selection

#### 5.3.1. Experimental Environment

This experiment aims to validate a framework that integrates viewpoint optimization using D-optimality with multi-robot-based manipulation task planning and execution.

In this study, we did not verify the extent to which the proposed method would improve performance when applied to actual collaborative tasks. To adequately handle the complexities of real cooperative manipulation tasks such as dynamic environments, complex object shapes, and recovery from grasping failures, it is essential to integrate not only visual assistance but also advanced motion planning, trajectory generation, and motion control. A comprehensive discussion of such integration is left for future work.

The focus of cooperation in this study is not on physical collaboration but rather complementary information sharing. This is an attempt to verify the principle effectiveness of visual support in collaborative manipulation tasks, and the complexity of the task is intentionally limited. Therefore, the problem setting we deal with here is a relatively simple manipulation, specifically object localization and two-point occupancy confirmation. This simplification is intended purely to evaluate the effectiveness of the proposed viewpoint selection method.

**Figure 10:**
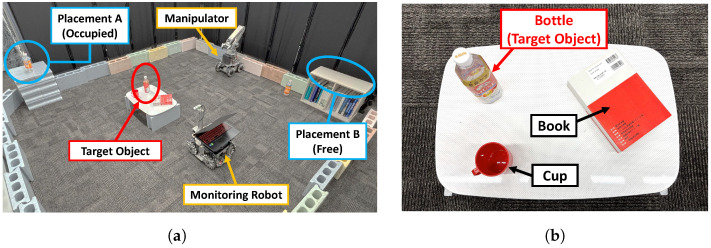
Setup for robotic manipulation experiments: (**a**) Overall configuration of the initial experimental environment. (**b**) Object placement on the table.

As shown in Figure 10a, the experimental environment consists of an indoor space partitioned by blocks and dividers. By placing blocks, we aim to improve the accuracy of environmental recognition using LiDAR and the stability of self-location estimation. There are two candidate placement locations. Placement location A is already occupied by a plastic bottle, while placement location B is vacant. The objective of the monitoring robot is to check the occupancy status of both locations and inform the manipulator of the position of placement location B.

In the experimental task, as shown in Figure 10b, a plastic bottle is preselected as the target object from three objects (a plastic bottle, a book, and a cup) placed on a table. The plastic bottle has dimensions of L×W×H=6cm×6cm×20.5cm. The plastic bottle was chosen as the target object because this study does not focus on shape estimation or grasping for complex-shaped objects. Additionally, the selected object has intermediate characteristics between a cuboid and a cylinder, meaning its appearance varies according to viewpoint, making it more generalizable than a purely cuboid object. The object is placed at (x,y,z)=(0m,1.5m,0.35m) in the manipulator’s base coordinate system.

The task assumed in this experiment involves the following steps: (1) The monitoring robot identifies the target object (plastic bottle) among the three objects on the table and searches for the optimal viewpoint by adjusting its viewing angle in approximately $10^{\circ }$ increments. (2) Based on the object information acquired by the monitoring robot, the grasping posture and the manipulator’s destination are determined, and manipulation is executed. The manipulator’s end effector has a maximum grip width of 9 cm, while the edge to be grasped is 6 cm. (3) After the manipulator completes grasping the target object, it requests a location from the monitoring robot for placement. The monitoring robot then checks the occupancy status of the two pre-identified locations. Based on the results, the monitoring robot provides the mobile manipulator with information on appropriate placement locations. The mobile manipulator subsequently transports and places the target object at the designated location, then the monitoring robot checks whether the task has been completed. These sequential actions are performed collaboratively by the robots, sharing information in real time to validate the effectiveness of collaborative manipulation based on optimal viewpoint selection.

D-optimality is a relative metric dependent on the characteristics of the environment and the object, and our results suggest that a viewpoint with high D-optimality in a particular environment will improve the accuracy of shape estimation. Therefore, to select the optimal viewpoint, it is necessary to predefine a threshold that can distinguish the relative fluctuations in D-optimality. The purpose of the preliminary evaluation in this experiment is not to determine the optimal viewpoint, but rather to understand how D-optimality varies depending on the viewpoint within the given environment, including its overall trend and distribution range. This allows for a reasonable assessment of the threshold setting for D-optimality to be used during the actual task execution.

**Figure 11:**
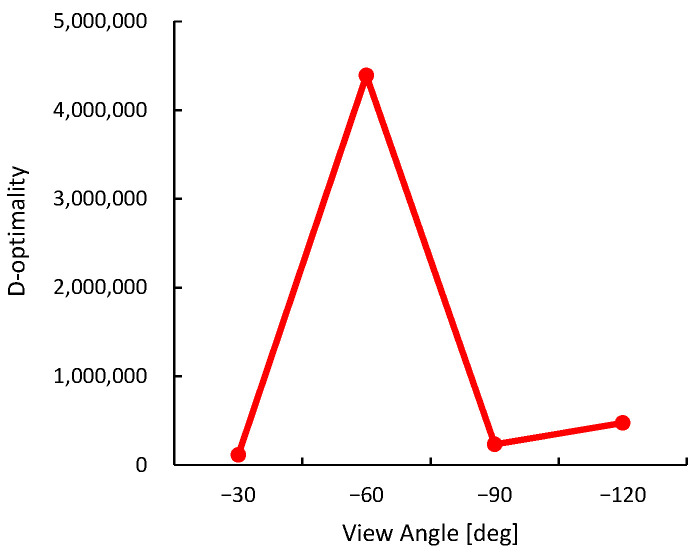
Preliminary evaluation of D-optimality in experimental environment.

For the preliminary evaluation of D-optimality in this experiment, shown in Figure 11, we collected 1000 3D data samples X of the object at four viewpoints $\alpha =0^{\circ },30^{\circ },60^{\circ },90^{\circ }$, and calculated D-optimality. Generally, objects with 3D shapes close to a cuboid are recognized as having similar shapes at 90 degree intervals from the viewpoint. Therefore, from the perspective of optimizing evaluation costs, four viewpoints from $0^{\circ }$ to $90^{\circ }$ were sampled for a sufficiently comprehensive preliminary evaluation. The preliminary evaluation only focuses on the plastic bottle, and other objects are completely uncertain for the monitoring robot during the task execution phase. The results show that D-optimality is significantly higher at $\alpha =30^{\circ }$ and relatively lower at other perspectives. This is consistent with previous basic verification results that D-optimality tends to rise sharply at specific viewpoints while remaining low at others. Considering exploration costs, the threshold was set to D=1,000,000 in this experiment.

On the other hand, during the actual task execution, the monitoring robot is required to dynamically explore the optimal viewpoint. To improve the accuracy of this exploration, the robot changes its viewpoint in $10^{\circ }$ increments. The difference in angular intervals between the preliminary evaluation and the task execution is due to their distinct objectives—capturing the overall distribution of D-optimality versus performing fine-grained optimization during exploration.

#### 5.3.2. Experimental Results

**Figure 12:**
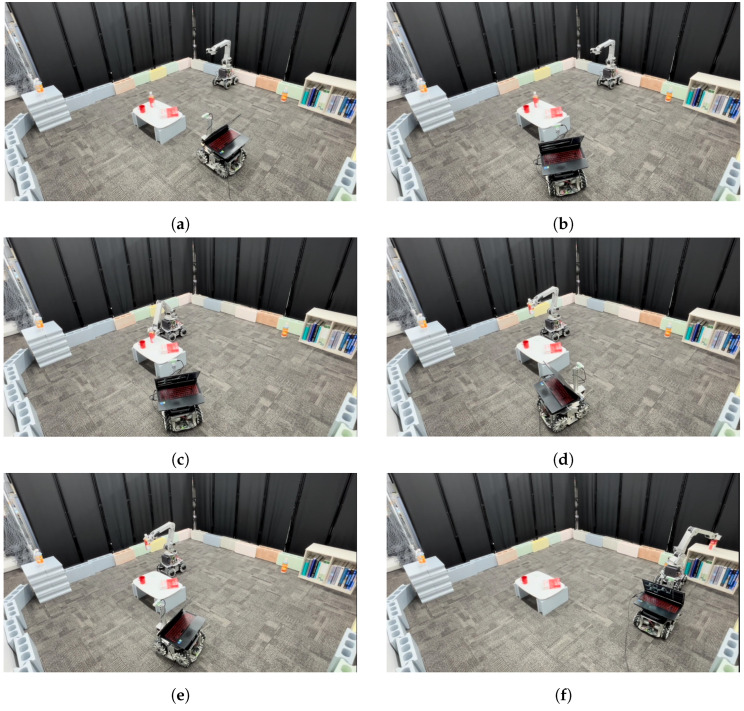
Snapshots of the multi-robot manipulation task: (**a**) Initial placement of each robot and object. (**b**) Optimal viewpoint selection by the monitoring robot and collaborative manipulation. (**c**) Object grasping and transportation by the mobile manipulator. (**d**) Confirmation of the occupancy status of placement location B. (**e**) Confirmation of the occupancy status of placement location A. (**f**) Object placement by the mobile manipulator and placement verification by the monitoring robot.

**Figure 13:**
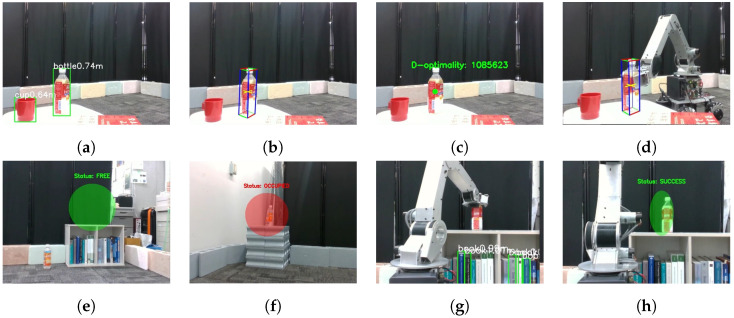
Snapshots(i.e., sequential visual observations) from the monitoring robot’s viewpoint during task execution: (**a**) Object recognition using YOLOv5. (**b**) Cuboid approximation of the target object. (**c**) Viewpoint evaluation based on D-optimality. (**d**) Grasping operation by the mobile manipulator. (**e**) Confirmation of occupancy status at placement location B (free). (**f**) Confirmation of occupancy status at placement location A (occupied). (**g**) Placement operation by the mobile manipulator. (**h**) Verification of object placement.

This experiment aims for the manipulator to grasp objects that it cannot detect independently and whose placement and shape are unknown, then transport them to a designated location from the monitoring robot. Figure 12a–f show the process of executing the cooperative manipulation task. Figure 13a–h show the sequence of viewpoints from the monitoring robot during task execution

Figure 13a–c show the monitoring robot’s viewpoint corresponding to the scene in Figure 12b. Figure 13a illustrates object detection using YOLOv5, where the detected object names and their respective distances are displayed. Figure 13b shows the cuboid approximation of the target object using CubeSLAM. Figure 13c presents the D-optimality value obtained from the viewpoint at $\alpha =-70^{\circ }$. The scene in Figure 12d corresponds to Figure 13e, and the scene in Figure 12e corresponds to Figure 13f, each showing the occupancy judgment results for the respective placement candidate positions. Finally, Figure 13h indicates that the monitoring robot successfully confirmed the completion of the placement task.

The monitoring robot then performs real-time three-dimensional shape estimation using CubeSLAM. It adjusts its viewpoint in approximately $10^{\circ }$ increments, autonomously evaluating each viewpoint by sampling 1000 shape estimation data points and assessing them using D-optimality.

In this experimental environment, viewpoints exceeding the threshold *D* = 1,000,000 were explored. The monitoring robot determined the optimal viewpoint at $\alpha =-70^{\circ }$, where *D* = 1,085,623. In Figure 12b, after moving to the optimal viewpoint, the monitoring robot shares object information in real-time with the mobile manipulator. As shown in Figure 12c, real-time information sharing was performed from the $\alpha =40^{\circ }$ viewpoint to execute object grasping and transportation. Figure 13d shows the visual information acquired by the monitoring robot. Subsequently, as shown in Figure 12f, the object was placed, completing the task.

The mobile manipulator set its target position at a reachable distance from the center of the target object, aligned vertically, and moved to that position. After completing the grasping operation, the monitoring robot determined the subsequent placement location. First, it confirmed that candidate location B was free, and then detected that candidate location A was already occupied. As a result, location B was selected. Subsequently, the mobile manipulator placed the object. After the placement operation, the monitoring robot verified the placement status and confirmed that the object had been accurately placed at the designated position. Through these procedures, the task was completed.

**Figure 14:**
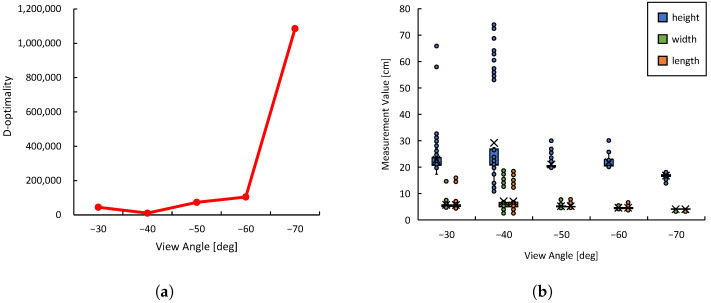
Data observed by the monitoring robot during task execution: (**a**) Transition of D-optimality. (**b**) Distribution of estimated object shape data.

In the experimental environment, viewpoints with a D-optimality value exceeding the threshold *D* = 1,000,000 are explored. Figure 14a shows the transition of D-optimality values across different viewpoints during task execution. At $\alpha =-70^{\circ }$, the D-optimality reached *D* = 1,085,623, based on which the optimal placement of the monitoring robot was determined.

As shown in Figure 14b, the manipulation was executed based on highly accurate and precise shape estimation results obtained from the viewpoint at $\alpha =-70^{\circ }$. In the following, we analyze the distribution characteristics of the shape estimation results from different viewpoints. At $\alpha =-30^{\circ }$ and $-40^{\circ }$, the distribution of the estimated values was wide and included numerous outliers. The information obtained from these viewpoints was unreliable in terms of both accuracy and precision, suggesting that sufficient shape information could not be acquired. Considering that the edge of the target object to be grasped is 6 cm long, while the maximum opening width of the manipulator’s end effector is 9 cm, manipulation based on the shape information from these viewpoints would likely have failed. On the other hand, at $\alpha =-50^{\circ }$ and $-60^{\circ }$, the variation in the data was relatively suppressed; however, the distribution remained wide, and outliers with errors of up to approximately 10 cm in the vertical direction were still observed. In contrast, at $\alpha =40^{\circ }$, the estimation results were highly reliable in terms of both accuracy and precision. Notably, the average error in the vertical direction was significantly reduced.

## 6. Conclusions and Future Work

In this study, we verified the effectiveness of a collaborative manipulation system based on viewpoint optimization using D-optimality. D-optimality is a metric that quantitatively evaluates the amount of information in observed data and is expected to contribute to improving measurement accuracy. In this study, we experimentally analyzed how changes in D-optimality influence object shape estimation accuracy and manipulation accuracy, and demonstrated its effectiveness. First, we verified the relationship between D-optimality and object estimation accuracy. As a result, we confirmed that viewpoints with high D-optimality have smaller variance in the acquired data, leading to improved shape estimation accuracy. In particular, at the viewpoint with the highest D-optimality, estimation error variance was reduced, enabling more stable shape estimation. Conversely, viewpoints with low D-optimality showed a tendency for larger estimation errors, indicating that viewpoint selection significantly impacts shape estimation accuracy. Next, we verified the impact of viewpoint selection based on D-optimality on manipulation accuracy. By evaluating the variation in the grasping positions of the manipulator’s end effector, we confirmed that viewpoints with high D-optimality resulted in improved object center coordinate estimation accuracy, which in turn enhanced grasping accuracy. Specifically, at the viewpoint with the highest D-optimality, the variation in the grasping position of the end effector was smaller, leading to a higher success rate. On the other hand, at viewpoints with low D-optimality, object shape estimation was unstable, resulting in a decreased success rate in manipulation tasks. Finally, we applied viewpoint optimization based on D-optimality to multi-robot collaborative manipulation and examined the relationship between viewpoint selection and manipulation accuracy. The experimental results confirmed that by searching for the optimal viewpoint, the monitoring robot could autonomously determine the most suitable viewpoint for manipulation, allowing the manipulator to perform grasping actions with high precision. This demonstrated that viewpoint optimization using D-optimality contributes to improving the accuracy of manipulation tasks in multi-robot systems.

The outcome of this research is that we have presented a dynamic framework for optimal viewpoint selection that enables optimal information acquisition and provision. In the future, it will be necessary for each robot to actively extract and select the required information from the acquired visual data.

In this paper, the information required for the manipulation task was predefined as geometric parameters. However, in real collaborative tasks, it is necessary for the robot to autonomously select and weight the relevant parameters according to the specific circumstances of each task and optimize the movement accordingly.

Moreover, it is important to extend the D-optimality-based motion planning framework to handle situations where the optimal viewpoint cannot be obtained, such as by proactively selecting and moving toward the next-best candidate.

Prospects for development include the following:

(1) Autonomous decomposition and understanding of tasks to set the necessary parameters by the robot.
(2) Algorithms for parameter selection and weighting.
(3) Action planning to efficiently acquire optimal information.

One specific application scenario is cooperative picking operations within warehouses. By integrating object shape information acquired from different viewpoints by each robot, it is possible to accurately estimate the dimensions and placement of target objects, thereby optimizing grasping strategies.

## Data Availability

The data presented in this study are available on request from the corresponding author.
